# Rhinovirus-Induced Modulation of Epithelial Phenotype: Role in Asthma

**DOI:** 10.3390/v12111328

**Published:** 2020-11-19

**Authors:** Aubrey N. Michi, Michelle E. Love, David Proud

**Affiliations:** Department of Physiology & Pharmacology, Cumming School of Medicine, University of Calgary, Calgary, AB T2N 4Z6, Canada; aubrey.michi@ucalgary.ca (A.N.M.); melove@ucalgary.ca (M.E.L.)

**Keywords:** rhinovirus, epithelial cell, asthma, chemokines, metabolism, barrier function, airway remodeling

## Abstract

Human rhinoviruses have been linked both to the susceptibility of asthma development and to the triggering of acute exacerbations. Given that the human airway epithelial cell is the primary site of human rhinovirus (HRV) infection and replication, the current review focuses on how HRV-induced modulation of several aspects of epithelial cell phenotype could contribute to the development of asthma or to the induction of exacerbations. Modification of epithelial proinflammatory and antiviral responses are considered, as are alterations in an epithelial barrier function and cell phenotype. The contributions of the epithelium to airway remodeling and to the potential modulation of immune responses are also considered. The potential interactions of each type of HRV-induced epithelial phenotypic changes with allergic sensitization and allergic phenotype are also considered in the context of asthma development and of acute exacerbations.

## 1. Introduction

Asthma is a chronic inflammatory airway disease characterized by symptoms of variable airflow limitation as well as by airway hyper-responsiveness and several structural changes to the airways, collectively referred to as airway remodeling. Asthma affects ~300 million people worldwide, and the prevalence in both adults and children has been increasing [1]. If current trends continue, asthma will affect an additional 100 million people by 2025 [2].

Human rhinovirus (HRV) infections are the most common respiratory viral infections in humans. In healthy, normal individuals, such infections lead to the self-limiting disease known as the common cold, but in subjects with existing lower airway diseases, such as asthma, the impact of these infections may be much more serious. HRV infections are a major trigger of acute exacerbations of asthma [3,4]. Moreover, there is clear evidence that HRV-induced wheezing episodes in early childhood are a contributing factor to the development of asthma in susceptible individuals [5,6].

Human airway epithelial cells (HAE) are the primary site of HRV infection in both the upper and lower airways [7,8,9]. Despite one study suggesting that induction of mucus cell metaplasia increases rhinovirus infection [10], the majority of studies have shown that ciliated epithelial cells are the dominant cell type infected by HRV [11,12,13]. HRV infections do not cause any overt epithelial cytotoxicity [14,15,16], leading to the concept that the effects of HRV infections in the airways must be mediated by changes in the biology of HAE. In support of this, it is known that HRV infection of cultured HAE can cause the release of numerous proinflammatory mediators, host defense molecules, and growth factors [17,18,19,20,21], many of which can also be detected in increased amounts in airway secretions during in vivo clinical HRV infections [18,19,20,21,22].

HAE phenotype is altered in both children and adults with asthma [23,24]. Changes include increased goblet cells and mucus production, reduced expression of tight junction proteins and impaired barrier function, epithelial fragility, and altered gene expression profiles [25,26,27,28]. Phenotypic changes of HAE from asthmatic compared to normal children, including increased expression of cytokeratin 5, altered production of cytokines, and enhanced production of growth factors linked to airway remodeling are maintained in culture [24,29,30,31,32]. This altered HAE phenotype is thought to arise from repeated cycles of exposure to environmental stimuli, including viruses like HRV [23,33], and the interactions of this altered epithelial phenotype with surrounding structural and immune cells have been implicated as a major contributor to the pathogenesis of asthma [34,35].

Despite the central role of HAE in regulating airway homeostasis, and the impact of HRV infections on asthma, the effects of HRV infections on HAE phenotype have not been fully characterized. In this article, we will review our current understanding of the ability of HRV infections to, at least transiently, alter several aspects of epithelial phenotype, and will discuss what factors may lead to transient changes to become permanent.

## 2. Modulation of an Inflammatory Phenotype

HRV infection of human airway epithelial cells induces expression of a number of proinflammatory cytokines and chemokines that can lead to inflammatory cell recruitment and tissue damage. A limited number of chemokines, such as the neutrophil chemoattractant, CXCL8, can be triggered by direct interactions of HRV strains with their specific receptors, leading to early, replication-independent signaling via receptor associated kinases that can activate phosphatylinositol 3-kinase and mitogen activated protein kinases (MAPK), leading to CXCL8 induction [36,37,38]. As may be expected with replication-independent signaling, CXCL8 secretion in this early time period can also be induced by ultraviolet (UV)-treated HRV, which can still bind to its receptor but cannot replicate [36].

**Figure 1:**
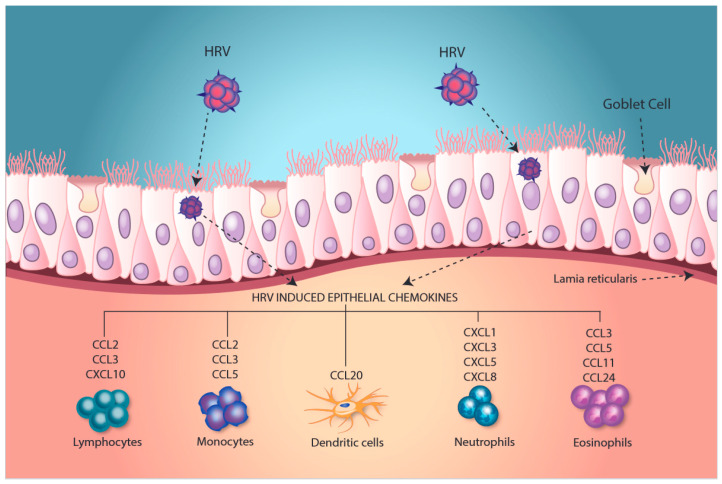
Human rhinovirus (HRV) infection of human airway epithelial cells can generate multiple chemokines that can recruit and/or activate numerous inflammatory cell populations.

Many more proinflammatory cytokines and mediators are induced in epithelial cells upon HRV infection via replication-dependent mechanisms. These include other cytokines that can support neutrophil recruitment to the airways, including both direct chemo-attractants, such as CXCL8, which can be induced by both replication-dependent and replication-independent pathways, CXCL1 and CXCL5 [39], and cytokines such as IL-17C, which can feedback onto epithelial cells to induce CXCL1 [40]. In addition, other chemokines, including but not limited to CXCL10, which is chemo-attractant for natural killer (NK) cells and some T cell subsets [41], and CCL5, which can recruit monocytes, NK cells [42], memory T cells [43], and eosinophils [44] (Figure 1) can be induced. These are absolutely dependent upon viral replication for HRV-induced secretion from HAE [19,45]. The dependence on viral replication has been linked to recognition of double-stranded RNA (dsRNA), an intermediate formed during HRV replication, by pattern recognition receptors, including the RNA helicases, RIG-I, and MDA-5 [46,47]. Toll-like receptor (TLR)3 has also been linked to recognition of rhinovirus dsRNA in HAE, but the presence of TLR3 in HAE is the subject of some controversy [46,47]. Moreover, blockade of TLR3 during experimental HRV infection in asthmatic subjects had no impact on chemokine production or symptoms [48]. Several studies have also suggested potential roles of other pattern recognition receptors in responses to HRV, including TLR2 and members of the NOD-like receptor (NLR) family of proteins [49,50,51,52]. Regardless of the specific pattern recognition receptor involved, downstream activation of NF-κB and of interferon regulatory factor (IRF) pathways occur, and these transcription factors are involved in regulating expression of many of the proinflammatory genes described above. In particular, among the IRF family, IRF-1 and IRF-7 have been shown to play a key role in gene regulation during HRV infections [53,54]. Although NF-κB and IRFs play a major role in cytokine production, other transcription factors, such as members of the STAT family of proteins [47], as well as MAPK pathways [55], also contribute to the regulation of replication-dependent genes.

While other cell types can also generate many of the same proinflammatory cytokines, it is important to note that many of the cytokines produced by epithelial cells in response to HRV infections are also detected in airway secretions during symptomatic rhinovirus infections [19,22,56,57]. It is known that increased levels of neutrophil-recruiting chemokines are detected during virus-induced asthma exacerbations, and that levels of such chemokines correlate with numbers of activated neutrophils and with the severity of asthma exacerbations [58,59]. There have been little data to convincingly support that HRV-induced chemokine production is markedly higher from asthmatic epithelial cells than from cells from normal subjects. It is likely, therefore, that, while inflammatory cell recruitment occurs in both subject groups, it is the occurrence of recruitment in the setting of existing airway inflammation and airway remodeling that leads to exacerbated lower airway disease in asthmatic subjects, while recruitment above a normal “zero” baseline does not cause lower airway symptoms in healthy individuals. Interactions between allergic and viral inflammation may also be a contributing factor.

## 3. Induction of Epithelial Antiviral Responses

As may be expected, HRV infection of HAE induces epithelial expression of a wide range of molecules with potential antiviral activity against a large number of viruses [60,61]. Again, the generation of antiviral genes is driven largely by viral replication-dependent pathways and via activation of both NF-κB and IRFs. The exact spectrum of which of these molecules specifically impact the pathogenesis of HRV infections still remains to be established, but several molecules have been shown to inhibit HRV replication and/or to modify aspects of the immune response to HRV. **V**irus **i**nhibitory **p**rotein, **e**ndoplasmic **r**eticulum-associated, **in**terferon-inducible (Viperin) is a protein that can affect replication of a number of viruses, and was shown to be among the most highly induced potential epithelial antiviral proteins upon HRV infection both in vivo and in vitro [60,61]. Knockdown of viperin in epithelial cells led to enhanced HRV replication, indicating a direct antiviral action of this protein against HRV [60]. Inducible nitric oxide synthase (iNOS) is the major enzyme generating nitric oxide (NO) in the airways. Not only is iNOS also induced in HAE in response to HRV infection [62], but NO directly inhibits HRV replication and also inhibits HRV-induced cytokine production [63,64]. Higher levels of exhaled NO during experimental HRV infections were associated with lower symptoms and more rapid viral clearance [65]. By contrast, interferon (IFN)-stimulated gene of 15kDa (ISG15) has no effect on HRV replication but is able to modulate HRV-induced RIG-I-mediated signaling to regulate the release of antiviral chemokines [21].

Given the established role of IFNs in regulating the response to many viruses, epithelial production of IFNs during HRV infection has been a topic of considerable investigation. At the protein level, HAE produce predominantly Type III (particularly IFN-λ1) IFNs and the Type I IFN, IFNβ [61,66]. It has been reported that HAE from asthmatic subjects have impaired production of both Type I and Type III IFNs [67,68], with subsequent studies indicating that IFN deficiency was not seen in well-controlled asthma [66], but was seen in severe, therapy-resistant asthmatic children [69]. The relative deficiency of IFN production in asthma remains controversial, however, with other studies finding no difference between asthmatics and normal subjects [70,71]. Moreover, no difference was seen between the levels of viral shedding from asthmatic and normal children with HRV infections [72]. It may be that reduced IFN levels are limited to subjects with severe asthma. If so, this would imply that IFN deficiency is a consequence of severe disease and not a predisposing factor for susceptibility to exacerbation in all asthmatics. In addition, a clinical trial using patients with moderate to severe asthma and a history of exacerbations after colds found that administration of IFNβ did not lead to any symptomatic improvement during infections [73]. This could be explained by an existing adequate level of IFNs to act as antivirals in all patients, despite variations in measured levels. On the other hand, there is ample precedent for antiviral molecules, including so-called interferon stimulated genes (ISGs) to be induced by IFN-independent pathways by multiple virus types [74,75,76], often by direct viral activation of transcription factors. Despite the clinical failure of IFNβ therapy, the concept of being able to use or induce direct antivirals that target HRV replication and signaling to mitigate HRV-induced exacerbations of asthma remains an attractive prospect. Although it has often been suggested that immune cells are required to clear HRV infections, recent data have shown that highly differentiated cultures of HAE are able to completely clear HRV infections [13], suggesting that modulation of epithelial antiviral defenses may be key to enhancing the rate of viral clearance.

## 4. Airway Remodeling and Altered Epithelial Barrier

The airways of asthmatic patients show a number of characteristic structural changes that are collectively referred to as airway remodeling. These include thickening of the lamina reticularis, increased airway smooth muscle mass, increased subepithelial matrix protein deposition, angiogenesis, and a marked change in the phenotype of the airway epithelium [23,77]. These remodeling changes are not congenital but can be observed in young, pre-school children, often before a formal diagnosis of asthma can be made [78,79]. Phenotypic changes observed in HAE include increased expression of cytokeratin 5, altered barrier function, increased mucus expression, altered production of cytokines and chemokines, and enhanced production of growth factors linked to airway remodeling [24,29,30,31]. Many of these changes appear to be epigenetic in nature as they are retained when cells are placed in the culture. Because these epithelial changes are already established by early childhood, it seems that they must be initiated by environmental exposures during this time period. Children experience 6–10 HRV infections per year [14], with a subset of children experiencing repeated episodes of HRV-induced wheezing illnesses [80]. It is this subset of children who are at increased risk of developing asthma [6]. Although it is not firmly established what renders only a subset of children susceptible to develop HRV-induced recurrent wheezing and subsequent asthma, one factor that seems to play an important role is the timing of HRV infections relative to allergic sensitization [81]. Since neither HRV induced wheezing alone, nor allergic sensitization alone guarantee asthma development and features of remodeling, the timing and nature of the interaction between these two processes appears critical. This raises the prospect, for example, that HRV infections alone may be able to cause transient changes in the epithelial phenotype but that appropriately timed allergic sensitization processes may be needed to transition these changes to a permanent phenotype. It is also feasible that, once a phenotype has been developed, subsequent HRV infections may enhance these changes as a part of induction of exacerbations.

There is ample precedent that HRV infection of epithelial cells can cause at least transient changes in several aspects of epithelial function. It is known that HRV infections can induce epithelial production, not only of cytokines and chemokines, as discussed above, but also of a number of growth factors and enzymes linked to various aspects of airway remodeling. These include members of the transforming growth factor-β family, including TGF-β and activin A, as well as members of the epidermal growth factor (EGF) and fibroblast growth factors families, such as amphiregulin, heparin-binding EGF, and fibroblast growth factor-2 [11,20], all of which have been linked to subepithelial fibrosis. HRV infected HAE also release a variety of extracellular matrix proteins that can contribute to subepithelial fibrosis [82] and enhanced airway smooth muscle responses [83], as well as matrix metalloproteinase-9 (MMP-9), which regulates matrix protein turnover [84]. HRV-infected HAE may also contribute to vascular remodeling and angiogenesis via production of vascular endothelial growth factor (VEGF) [20,85]. Both VEGF and active MMP-9 are present in increased amounts in airway secretions during HRV infections [20,84]. It is noteworthy that TGF-β and VEGF are both produced in increased levels from asthmatic versus normal epithelial cells stimulated with IL-13 or IL-4 [31], further supporting the potential interaction between HRV infections and allergic sensitization. Another feature of airway remodeling is an increase in the number of fibroblasts/myofibroblasts in the lamina propria. In addition, not only does airway smooth muscle mass increase, but smooth muscle is seen closer to the airway epithelium. Both smooth muscle cells and fibroblasts can play a role in regulating subepithelial fibrosis. It has been shown that supernatants from HRV-infected HAE are chemotactic for both fibroblasts and smooth muscle cells. This is due to an increased release of chemokines that are usually thought of as chemotactic for inflammatory cells. It has been shown that chemoattraction of fibroblasts by HRV-infected epithelial cells is due to the release of CXCL10 and, to a lesser extent, CXCL8 [86]. By contrast, recruitment of smooth muscle cells depends on viral production of CCL5 [87]. Thus, HRV infections could help induce thickening of the lamina reticularis by recruiting structural cells closer to the epithelial layer to deposit matrix proteins.

Increased mucin gene expression and mucus metaplasia is also a feature of asthmatic airway epithelium. Allergic sensitization may again play a role in this phenomenon, as IL-13 is a major inducer of mucus metaplasia and of the major airway mucin gene, MUC5AC, in HAE [10,11]. Again, the potential for possible interactions between allergic sensitization and HRV infections arises, as several studies have shown that HAE infected with HRV produced increased expression of MUC5AC [88,89,90]. This induction depends upon activation of the EGF receptor and downstream induction of the ERK1/2 MAPK pathway and of NF-κB [88,89]. Increased MUC5AC expression was also observed in the airway epithelium of both normal and asthmatic subjects during experimental HRV infections [89].

A major feature of the asthmatic epithelium is a decrease in barrier function that may underlie the epithelial fragility characteristic of the disease. Barrier function of HAE is controlled primarily at the level of tight junctions. Ultrastructurally, tight junctions appear as an interconnected belt-like network of linearly arranged strands that encircle the cell. Almost 40 different proteins have been identified as components of tight junctions [91]. These include integral junctional proteins, such as occludins, junctional adhesion molecule (JAM)-1, and members of the claudin family of proteins. These proteins bridge the intercellular space and help to regulate epithelial permeability, and macromolecular transport. The C-terminus of claudins contain binding sites for junctional plaque proteins, such as the members of the zona occludens family, ZO-1, ZO-2, and ZO-3, that link the intercellular space proteins to the cytoskeleton. Finally, a number of cytosolic and nuclear proteins, such as cingulin, symplekin, and the phosphatase and tensin homolog (PTEN), can interact with the plaque proteins to regulate several aspects of cell function.

Although one publication reported that HRV-16, which uses ICAM-1 as its cell surface receptor, did not induce any loss of epithelial barrier function [10], two reports showed a transient loss of barrier function, as assessed by loss of tight junction proteins and reduced transepithelial resistance, when epithelial cultures were exposed to HRV-1B, which uses members of the low density lipoprotein (LDL) receptor family for cell entry [33,92]. We have recently compared the effects of equal genome copy numbers of HRV-16, HRV-1A, and HRV-C15 on epithelial expression of ZO-1 and occludin, and on barrier function assessed by leakage of FITC-dextran. In agreement with earlier studies, we found little effect of HRV-16 on barrier function but saw clear effects with both HRV-1A and HRV-C-15 with the latter causing the greatest effect (Michi, A.N., Proud, D.—unpublished data). These effects were dependent upon viral replication. The failure of HRV-16 to show any measurable responses may be related to the fact that major group viruses can only infect 5–10% of primary HAE, regardless of the infectious dose used [9], presumably due to the limited expression of ICAM-1 on epithelial cells. By contrast, minor group HRV strains can infect a substantially higher percentage of cells due to a wider expression of LDL receptors [93]. HRV-C15 uses cadherin related family member 3 (CDHR3) to gain cell entry and this molecule is expressed on up to 80% of ciliated cells in highly differentiated HAE [12].

The mechanisms by which HRV infection regulates the barrier function of HAE remains to be fully determined. Studies to date have examined regulation at 24 h after infection and demonstrate a dependence, at least for HRV-1B, on NOD-like receptor X-1 recognition and downstream activation of NADPH oxidase 1-mediated generation of reactive oxygen species (ROS) [52,94]. Migration of NLRX-1 to the mitochondria was also observed along with activation of mitochondrial ROS, and blockade of ROS was shown to regulate changes in transepithelial resistance, although other indices of barrier function were not studied [52].

It must be noted that all studies in cells from normal individuals show that HRV infection causes a transient loss of epithelial barrier function. It is unclear whether this is due to reversal of viral replication-mediated signaling, or to shedding of infected or damaged cells and repair of the epithelial structure by spreading or differentiation of progenitor cells. Clearly, additional stimuli must play a role in converting these transient alterations of phenotype to the permanent changes observed in asthma. It is of interest, however, that, once the asthmatic phenotype is induced, HRV infections can cause a more prolonged effect on barrier function in cells from asthmatic patients [92].

## 5. Modulation of Immune Interactions

There is considerable evidence that human airway epithelial cells can interact with cells of the innate and adaptive immune systems to regulate their functional responses. Airway epithelial cells are sources of the cytokines IL-33, IL-25, and thymic stromal lymphopoietin (TSLP). HRV infections have been reported to lead to increased production of these cytokines, either in vitro or in vivo [95,96,97]. Each of these cytokines are linked to activation of innate lymphoid type 2 cells (ILC2) leading to increased Th2-type allergic inflammation. In addition to impacting ILC2 cells, epithelial cells may also modify dendritic cell recruitment and function. HRV induces expression of epithelial cell CCL20 and human β-defensin 2 (HBD2) [18,98], which are ligands for the CCR6 receptor found on immature dendritic cells. Attracting these cells to the airways would enhance antigen surveillance and capture. HRV infected epithelial cells can differentially regulate dendritic cell function dependent upon the balance of epithelial products produced in a given scenario. HRV infections induce production of IL-1β, IL-6, and IL-15 [22,99,100], which all help promote dendritic cell maturation. By contrast, viral induction of VEGF and NO would be expected to inhibit dendritic cell maturation [101]. In addition to its effects on ILC2 activation, HRV-induced TSLP activates and polarizes dendritic cells to a phenotype that drives CD4^+^ T cells to Th2 differentiation that would support development of allergic inflammation [102]. The ability to regulate ILC2 and dendritic cell function raises the question of whether repeated HRV infections in childhood, in conjunction with exposure to allergic stimuli may help to polarize the immune response to a Th2 bias. This could contribute to the observation that rhinovirus-induced wheezing episodes in early childhood predispose children to asthma development [5,81].

Human airway epithelial cells constitutively express cell surface major histocompatibility complex (MHC) class I molecules, but this expression is enhanced upon rhinovirus infection [103]. Epithelial cells also constitutively express members of the B7 family of costimulatory molecules, including B7-H1, B7-H2, B7-H3, and B7-DC [104]. HRV infection, or stimulation with IFNγ, upregulates expression of B7-H1 and B7-DC [104,105]. Blocking the activity or either of B7-H1 or B7-DC led to increased T cell activation and production or IFNγ in co-culture experiments, suggesting that these B7 homologues are inhibitory [104]. If suppression of the Th1 cytokine IFN is selective, this may also favor induction of a Th2 environment. Interestingly, HRV infection of epithelial cells increases expression of the rhinovirus receptor, ICAM-1. Interaction of epithelial ICAM-1 with lymphocyte function-associated antigen 1 (LFA-1) on T cells increases lymphocyte proliferation and stimulates production of Th2 cytokines [106]. Thus, while antiviral immunity is normally considered to be dominantly of the Th1 phenotype, under appropriate conditions, it is feasible that repeated HRV infections in childhood can function in concert with antigen exposure to enhance a Th2 environment in the airways of children that could facilitate the development of asthma.

## 6. Summary and Implications for Asthma Pathogenesis

It is now well recognized that the airway epithelium plays a major role in regulating many aspects of airway homeostasis and that altered epithelial function can contribute to the pathogenesis of airway diseases [107]. As noted above, HRV infections can alter multiple aspects of airway epithelial function in ways that are relevant both for the development of asthma and for acute exacerbations of the disease.

**Figure 2:**
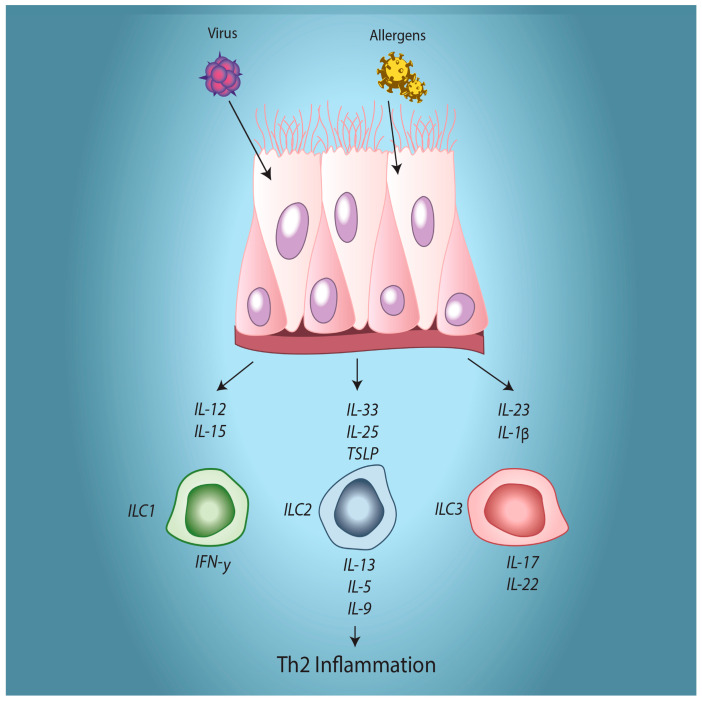
Human rhinovirus (HRV) infection and allergic stimulation can both induce epithelial production of IL-33, IL-25, and thymic stromal lymphopoietin (TSLP) that will stimulate activation of ILC2 cells to drive Th2 inflammation.

Although virtually all young children experience multiple HRV infections annually, only a subset of children develop episodes of HRV-induced wheezing illness. A similar paradox has also been reported for respiratory syncytial virus (RSV)-induced wheezing illness, which has also been linked to asthma development [108]. In a high-risk birth cohort, children who experienced HRV-induced wheezing episodes in the first three years of life are about 10 times more likely to subsequently develop asthma compared to those who did not have wheezing illnesses [6]. Interestingly, RSV-induced wheezing increased the odds or asthma development by a factor of only 2.6 and those who had wheezing episodes linked to both viruses did not show any significantly increase risk above those with HRV-induced wheezing alone [6]. Those children with multiple viral wheezing episodes seem particularly likely to develop asthma [109]. It remains unclear what factors distinguish those children who develop virus-induced wheezing episodes, but multiple factors including genetic predisposition, allergic sensitization, impaired lung development, second-hand cigarette smoke exposure, antibiotic use, and attendance at daycare have all been suggested to regulate susceptibility to wheezing and asthma development [110]. A key issue in terms of the impact of environmental factors in asthma development is likely to be the timing of exposures. Virus-induced wheezing and allergic sensitization are independent risk factors for the development of asthma [6]. Although a study in a high-risk birth cohort found that asthma development was more likely if allergic sensitization preceded virus-induced wheezing, this was not an absolute relationship, as more children experienced virus-induced wheezing in the first year of life than developed allergic sensitization [81]. While the relative timing of these independent variables requires additional study, there is ample rationale for the interaction between these two stimuli. The majority of asthma cases are associated with a Th2 inflammatory milieu, as would be expected with allergic sensitization, IgE class-switching, and T cell polarization. However, as discussed above, HRV infection could enhance these processes in several ways. Viral release of epithelial chemokines and cytokines can recruit dendritic cells and T cells to the airway. Epithelial generation of TSLP, IL-25, and IL-33 would be expected to impact ILC2 cells to enhance Th2 inflammation (Figure 2), while TSLP can also polarize dendritic cell function to enhance a Th2 environment. In addition, costimulatory molecules expressed by HRV-infected epithelial cells can also help to drive T cells to a Th2 phenotype. HRV-infected epithelial cells could also contribute to asthma development by stimulating multiple aspects of airway remodeling. We now know that these processes can begin in early childhood, and it is likely that allergic inflammation may interact with HRV infections to enhance or sustain these processes. We know that epithelial cells from asthmatic children differentially produce remodeling factors, regulate matrix protein deposition, and show a differential fibroblast to myofibroblast transition [31,32,111], but additional studies are required to better define the interactions between allergic exposure and HRV infection both in causing permanent epithelial phenotype changes and in regulating growth factor production.

Once asthma has developed, HRV infections can trigger acute exacerbations in asthmatics of all ages. In several studies from some countries, such as Australia and Finland, HRV-C strains have been associated with severe exacerbation in young children [112,113,114], while, in the US, both HRV-C and HRV-A strains have been linked to a more severe disease outcome [114,115]. The pathogenesis of HRV-induced exacerbations in asthma is not well understood but they are not well-controlled using standard asthma therapies. Multiple studies have confirmed increases in inflammatory mediators in the lower airways in asthma. Since HRV infections do not cause lower airway diseases in healthy subjects, it is reasonable to suggest that a combination of virus-induced inflammation together with pre-existing allergic inflammation in the setting of airway remodeling leads to exacerbation. This is consistent with the observation that exacerbations do not occur as frequently in patients whose baseline asthma is well controlled. One can envision that the levels of virus-induced inflammation are regulated by two main parameters: the degree of virus induction of proinflammatory mediators, and the host antiviral response occurring in response to HRV infection. Thus, one potential therapeutic approach would be to block the actions of inflammatory mediators induced by HRV infection. It is unclear, however, which specific mediators should be targeted for inhibition, as some mediators, such as CXCL10, can recruit natural killer cells and some lymphocyte subtypes to the airways that may also contribute to innate immunity and viral clearance. Additional studies are needed to address this issue and to examine potential interactions between viral and allergic inflammation. An alternative approach to therapy would be to strengthen host antiviral defenses. Although attempts to enhance the global antiviral response could be considered, it is likely that a targeted approach to modulate key antivirals selective for HRV may be preferable. As noted above, it has been suggested that impaired IFN induction in asthmatic subjects during HRV infections may permit exacerbations, but IFN treatment failed to have any significant effect [73]. It is known that NO both inhibits HRV replication in airway epithelial cells and also suppresses virus-induced cytokine production [63,64]. Moreover, during experimental HRV infections, patients who produced the highest levels of exhaled NO had lower symptom scores and cleared the virus more rapidly [65]. Thus, topical administration of NO donor compounds may represent a potential approach to enhancing antiviral immunity to HRV infections. Further studies are required to fully identify the spectrum of key antivirals during HRV infections to determine which molecules provide potential targets for therapeutic interventions during asthma exacerbations.
